# Domain II of *Pseudomonas* Exotoxin Is Critical for Efficacy of Bolus Doses in a Xenograft Model of Acute Lymphoblastic Leukemia

**DOI:** 10.3390/toxins10050210

**Published:** 2018-05-21

**Authors:** Fabian Müller, Tyler Cunningham, Richard Beers, Tapan K. Bera, Alan S. Wayne, Ira Pastan

**Affiliations:** 1 Laboratory of Molecular Biology, Center for Cancer Research, National Cancer Institute, National Institutes of Health, Bethesda, MD 20892, USA; fabian.mueller@uk-erlangen.de (F.M.); cunningham.tyler@gmail.com (T.C.); beersr@dc37a.nci.nih.gov (R.B.); berat@mail.nih.gov (T.K.B.); 2 Department of Hematology/Oncology, University Hospital Erlangen, Erlangen 91054, Germany; 3 Children’s Center for Cancer and Blood Diseases, Division of Hematology, Oncology and Blood and Marrow Transplantation, Children’s Hospital Los Angeles, Norris Comprehensive Cancer Center, Keck School of Medicine, University of Southern California, Los Angeles, CA 90027, USA; awayne@chla.usc.edu

**Keywords:** immunotoxin, Moxetumomab pasudotox, targeted therapy, CD22, B cell non-Hodgkin lymphoma, acute lymphoblastic leukemia, mantle cell lymphoma

## Abstract

Moxetumomab pasudotox is a fusion protein of a CD22-targeting antibody and *Pseudomonas* exotoxin. Minutes of exposure to Moxetumomab achieves similar cell killing than hours of exposure to a novel deimmunized variant against some acute lymphoblastic leukemia (ALL). Because blood levels fall quickly, Moxetumomab is more than 1000-fold more active than the deimmunized variant in vivo. We aimed to identify which part of Moxetumomab increases in vivo efficacy and generated five immunotoxins, tested time-dependent activity, and determined the efficacy in a KOPN-8 xenograft model. Full domain II shortened the time cells had to be exposed to die to only a few minutes for some ALL; deimmunized domain III consistently extended the time. Against KOPN-8, full domain II accelerated time to arrest protein synthesis by three-fold and tripled PARP-cleavage. In vivo efficacy was increased by more than 10-fold by domain II and increasing size, and therefore half-life enhanced efficacy two- to four-fold. In summary, in vivo efficacy is determined by the time cells have to be exposed to immunotoxin to die and serum half-life. Thus, domain II is most critical for activity against some ALL treated with bolus doses; however, immunotoxins lacking all but the furin-cleavage site of domain II may be advantageous when treating continuously.

## 1. Introduction

Therapeutic antibodies have become the standard of care in the treatment of a variety of diseases, including cancer [1]. Therapeutic antibodies enable a specific attack against target cells, while the unrelated tissue remains unaffected. To further increase the efficacy of therapeutic antibodies, the constant fragment (Fc) of antibodies has been engineered to improve recruitment of complement and immune cells [2] and antibodies were armed with radionucleotides [3], chemotherapeutic drug conjugates [4,5], or plant and bacterial toxins [6]. The latter, also called immunotoxins, show high clinical activity against hematologic malignancies [6,7]. The immunotoxin Moxetumomab pasudotox consists of a CD22-targeting disulfide-stabilized antibody fragment (dsFv) and a 38 kDa fragment of *Pseudomonas* exotoxin (PE). Moxetumomab pasudotox (hereafter referred to as dsFv-PE38) produces response rates of 86% in patients with relapsed/refractory (r/r) hairy cell leukemia [8], and of 32% in r/r pediatric acute lymphoblastic leukemia (ALL) [9].

Because patients that are treated with immunotoxins often develop anti-drug antibodies which likely reduce activity [8,10], less immunogenic immunotoxins have been developed. Major B- and T-cell epitopes that were located in domain II of *Pseudomonas* exotoxin were removed by deletion of all but the furin cleavage site [11] resulting in a 24 kDa PE (PE24) which on average is 2-fold more active than PE38 [12]. To further reduce immunogenicity, seven mutations that disrupt B-cell epitopes were introduced in domain III, hereafter referred to as PE24(B) [13,14]. By extending the 25 kDa dsFv of PE24(B) to a 50 kDa Fab, serum half-life and activity of bolus doses greatly increased [14]. Fab-PE24(B) achieved sustained complete remissions in a subcutaneous CA46 xenograft model; however, dsFv-PE38 could not.

Opposite the CA46 model, a systemic xenograft with the ALL cell line KOPN-8 responded substantially better to dsFv-PE38 than to Fab-PE24(B) [15]. The KOPN-8 bone marrow (BM) infiltration was more than 1000-fold more efficiently reduced after five doses of the maximally tolerated dose (MTD) of 0.4 mg/kg dsFv-PE38 QOD than after five doses of the MTD of 2.0 mg/kg Fab-PE24(B) [15]. We showed that the reason for the substantial difference in the efficacy was the time that KOPN-8 cells had to be exposed to immunotoxin to die. While the cells had to be exposed to dsFv-PE38 for only a few minutes to induced more than 50% cell death, they had to be exposed to Fab-PE24(B) for more than 6 h in order to reach similar cytotoxicity. Because the serum concentration of immunotoxin after a bolus dose fall within hours to inactive levels, bolus doses of Fab-PE24(B) did not reduce KOPN-8 tumor burden in vivo.

Here, we determine which part of dsFv-PE38 is the most critical to achieve the 1000-fold stronger efficacy in the KOPN-8 animal model. Five distinct immunotoxin variants were generated and were tested for their time-dependent activity against various B-cell malignancies and patient-derived ALL blasts in vitro, and findings that were validated in the systemic KOPN-8 xenograft model.

## 2. Results

### 2.1. Wild-Type PE24 Immunotoxin Shows Highest Overall Activity

**Figure 1:**
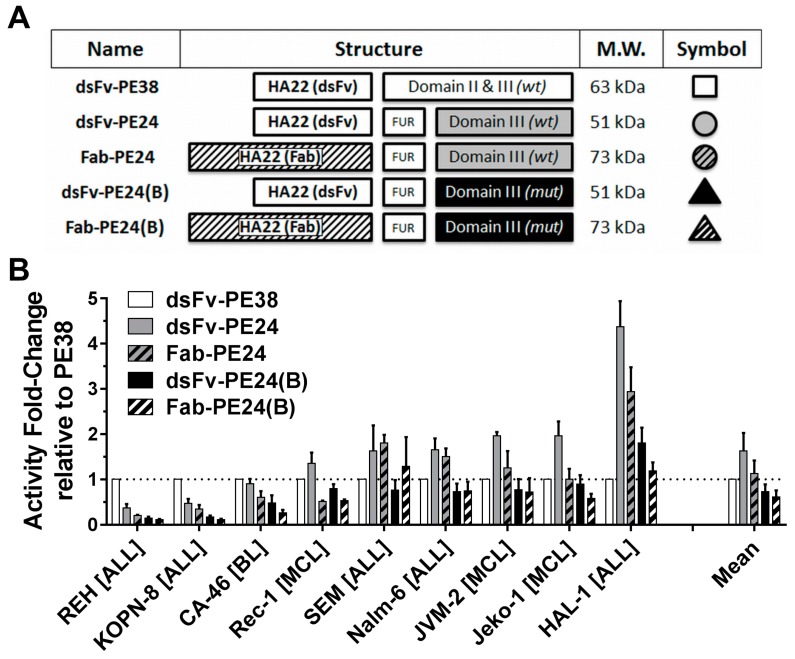
Variants of CD22-targeted immunotoxins are active in vitro. (**A**) Cartoon depicting the differences among five immunotoxin variants. Indicated are the names of the molecules, the antibody structure as disulfide-stabilized Fv (dsFv) or Fab, the respective PE-structure either as wild-type (WT) PE or a PE containing B-cell epitope-depleting mutations (mut) [14], the respective molecular weight (MW), and the symbol/color-code used throughout the manuscript; (**B**) The activity (1/IC_50_) of the indicated immunotoxin variants against various malignant B-cell lines was determined by WST8. Shown are the average fold-changes relative to dsFv-PE38 of at least three independent experiments, errors as standard error of mean (SEM).

To test which protein domain was responsible for the substantial difference of activity of dsFv-PE38 and Fab-PE24(B) against the KOPN-8 cell line, we constructed a total of five molecules controlling for the three major differences, namely size of the antibody fragment, full domain II versus furin cleavage site, and mutated domain III versus wild-type (WT) domain III (Figure 1A). By removing all but the furin cleavage site of domain II of the WT PE38, the molecule dsFv-PE24 was generated [12], and by exchanging the dsFv with a Fab, the novel 23 kDa larger Fab-PE24 was achieved. By replacing PE24 with PE24(B), the two immunotoxins dsFv-PE24(B) and Fab-PE24(B) were generated [14]. Various B-cell malignancy cell lines were continuously treated for three days with immunotoxin, and the concentration at which 50% of cell growth was inhibited, as determined by the WST-8 assay (Supplementary Table S1). With an IC_50_ of 0.3 pM against the Reh cell line, dsFv-PE38 was the most active of the five immunotoxins. The dsFv-PE24 and Fab-PE24, however, showed the highest average activity overall cell lines with average IC_50_s of 6.9 pmol/L and 10.9 pmol/L, respectively. In addition to highest average activity, the PE24 variants showed the least variability with an approximately a three-fold lower variance (σ²) than PE38 or PE24(B) variants. We mathematically determined the fold-difference of cytotoxicity of the immunotoxin variants relative to dsFv-PE38 (Figure 1B). The larger Fab-containing immunotoxins were generally less cytotoxic in vitro than their respective smaller, dsFv-containing molecule. This finding was consistent for both WT and the mutant PE24. Against the Reh and the KOPN-8 cell line, dsFv-PE38 was more active than any other molecule, while for the other cell lines, WT PE24 without domain II was the most active. Confirming previous results [14], the activity of mutated PE24(B) was, on average, similar to that of dsFv-PE38 in this assay.

### 2.2. Immunotoxin Variants Show Highly Variable Time to Reach Maximal Cytotoxicity

**Figure 2:**
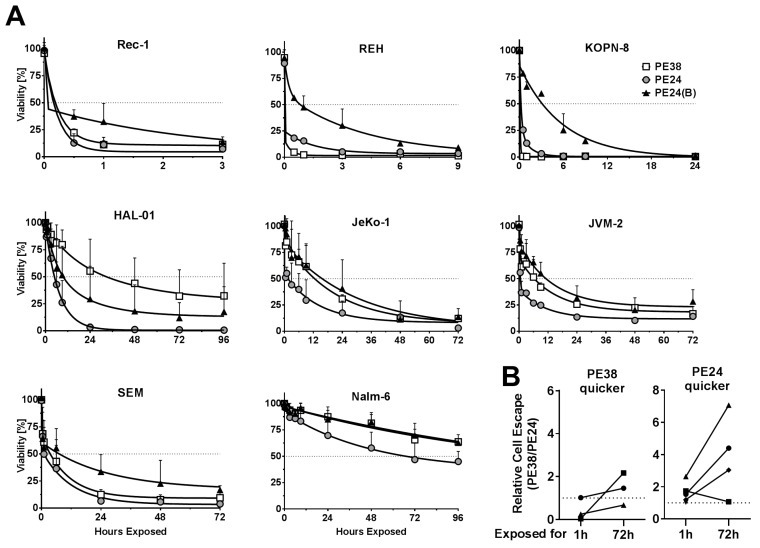
Immunotoxin variants induce exposure time-dependent cell death. (**A**) Indicated B-cell lymphoma cell lines were treated with 2.8 nmol/L immunotoxin for the indicated times, washed, and replated. Three days after assay initiation, cell viability was determined by flow cytometry. The symbols indicate the mean % of living cells at each data point of at least three independent experiments, errors are shown as SEM, curve fitting was done using two-phase decay regression analysis using GraphPad; (**B**) The rate of relative cell escape was determined as % surviving cells after PE38/% surviving cells after PE24 after 1 h and 72 h of drug exposure, respectively. Cell lines from A were divided into two groups, one group showing less escape after 1 h of PE38 (PE38 quicker) and one group showing less escaping cells after 1 h of PE24 (PE24 quicker). Results of the same cell line after 1 h and after 72 h of exposure are connected by lines.

Previously, we found that cells have to be exposed to CD22-targeted immunotoxins for a highly variable time for them to die [15]. Accordingly, we tested the time cells that had to be exposed to the five immunotoxins to induce cell death. We chose an equimolar concentration of 2.8 nmol/L immunotoxin, because it correlates with the amount of Fab-PE24(B) that reaches KOPN-8 cells growing in the murine BM after an intravenous single dose [15]. After treating with 2.8 nmol/L, the cells were washed at indicated times, replated in fresh medium, and three days after start of the assay, enough time for a cell that was exposed to a lethal dose of immunotoxin to die, cell viability was determined. The time that is needed to reach more than 50% cell death varied widely from less than 30 min to more than four days (Figure 2A, Supplementary Table S2). For the Reh and the KOPN-8 cells, the PE38-variant needed much less exposure time than the PE24 or the PE24(B) variants to induce cell death. All of the other cell lines were killed after shorter exposure time to the WT PE24 than to the mutated PE24(B) or the domain II-containing PE38. This was most pronounced for HAL-01, where the PE24-variant induced more than 50% cell death after 6 h, the PE24(B) after 9 h, and the PE38 after 24 h, suggesting that full domain II increased the exposure time needed to kill HAL-01 cells by 4-fold. Cells consistently had to be exposed longer to the PE24(B) variants than to WT PE24 to induce cell death. As suggested by the slightly higher activity in WST-8 assays, the dsFv-immunotoxins always needed shorter exposure time than their respective Fab variant to reach similar cell killing (Supplementary Figures S1 and S2). Whether an immunotoxin variant was more cytotoxic than any other variant after 1 h of exposure did not predict whether it remained as the most active variant after 72 h of exposure. To enable a comparison, we mathematically generated the relative cell escape as the ratio of living cells after treatment with two distinct immunotoxins. Comparing relative cell escape showed that, even though being slower initially, treatment with Fab-PE24 was more potent than dsFv-PE38 at later time points against most cell lines (Figure 2B). Cell lines, which showed less cell escape after 1 h of treatment with Fab-PE24 than after dsFv-PE38, consistently showed less escaping cells when being treated with Fab-PE24 for 72 h. On the other hand, two of three cell lines showing less relative cell escape after 1 h of treatment with dsFv-PE38 than with Fab-PE24 showed less escaping cells after 72 h of exposure to Fab-PE24 than to dsFv-PE38.

### 2.3. In Vivo Efficacy Emphasizes Importance of Domain II against KOPN-8

**Figure 3:**
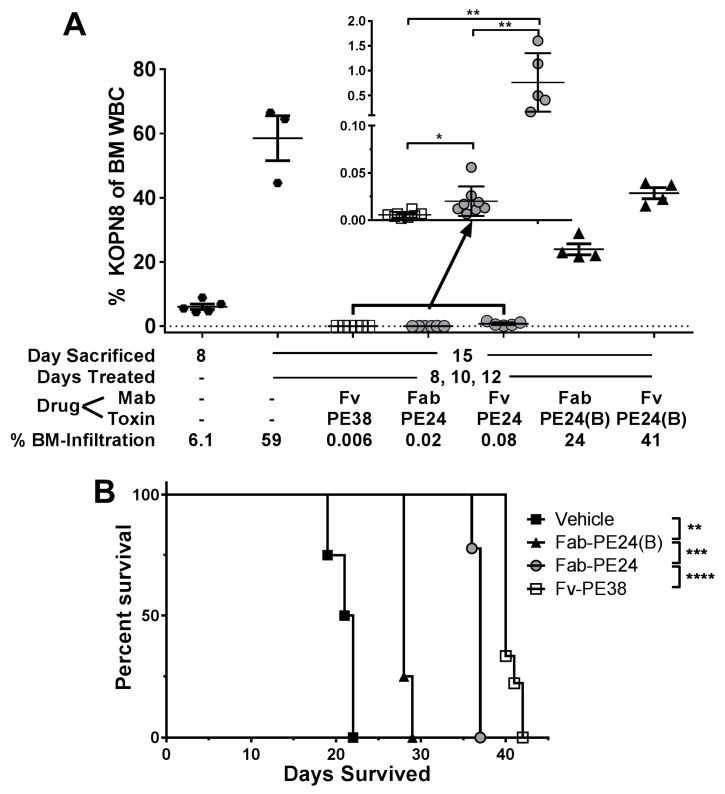
CD22-targeted immunotoxin variants show highly variable efficacy in vivo. (**A**) Mice that were intravenously injected with KOPN-8 on day 1 were either sacrificed on day 8 or treated with the indicated immunotoxin on days 8, 10, and 12 and sacrificed on day 15. BM was extracted and analyzed for KOPN-8 BM infiltration. Each symbol represents an individual mouse, bars indicate the mean of the respective group, errors are shown as SEM, *p*-values were determined by unpaired *t*-tests as * < 0.05 and ** < 0.01; (**B**) KOPN-8-bearing mice were treated with three doses of the indicated immunotoxin or vehicle and followed until disease progression. Shown is a Kaplan-Mayer plot, *p*-values determined by log-rank tests as ** < 0.01, *** < 0.001, and **** < 0.0001.

We tested which of the molecular differences of dsFv-PE38 and Fab-PE24(B) most critically reduced the efficacy of intravenous bolus doses of immunotoxins. KOPN-8-bearing mice were treated with five doses immunotoxin at their MTD QOD and KOPN-8 BM infiltration rate was determined three days after the last dose (Figure 3A). Mice before treatment at day 8 showed, on average, a KOPN-8 BM infiltration of 6.1%, which rose to 59% at day 15 in untreated mice. Five doses of 0.4 mg/kg dsFv-PE38 resulted in 0.006%, of 1.0 mg/kg Fab-PE24 in 0.02%, of 2.0 mg/kg dsFv-PE24 in 0.08%, of 2.5 mg/kg Fab-PE24(B) in 24%, and of 4.0 mg/kg dsFv-PE24(B) in 41% KOPN-8 BM infiltration at day 15. Similar to previous results, five bolus doses of dsFv-PE38 reduced KOPN-8 BM infiltration 4000-fold more efficiently than Fab-PE24(B). Because the Fab-variants produced better responses than the dsFv variants in vivo, we focused on Fab-PE24, Fab-PE24(B), and dsFv-PE38, and tested next their effects on the survival of KOPN-8 bearing mice (Figure 3B). In line with the rate of reduction of KOPN-8 BM infiltration early after treatment, the median animal survival of KOPN-8 bearing mice treated with vehicle was 21.5 days, with Fab-PE24(B) 28 days, with Fab-PE24 37 days, and with dsFv-PE38 40 days.

### 2.4. Biochemical Events Reflect Differences in Immunotoxin Activity

**Figure 4:**
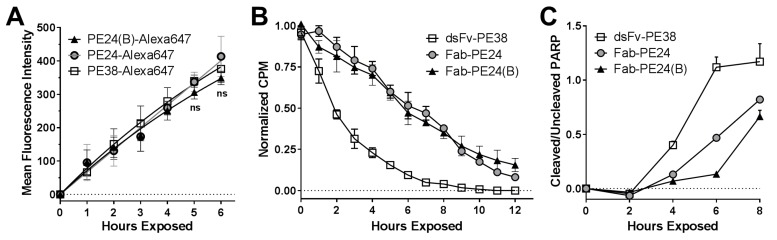
Differences of immunotoxin activity is reflected by distinct biochemical events. (**A**) The rate of internalization of immunotoxin molecules into KOPN-8 cells was determined by flow cytometry. The graph summarizes the mean fluorescence intensity of Alexa647 over time from three independent experiments, each done in triplicates, errors as SEM, significance determined by *t*-tests. The mean fluorescent intensity was corrected for the relative amount of Alexa647 conjugated with the respective immunotoxin variant; (**B**) Protein synthesis was quantified by [^3^H]-leucine-incorporation, whereas the counts per minute (CPM) after treating KOPN-8 cells with immunotoxin for the indicated times were measured following an additional 1 h [^3^H]-leucine pulse. Each data point was acquired in quadruplicates and the graph summarizes two independent experiments; errors shown as SEM; and, (**C**) The levels of loading control and whole and cleaved PARP were detected from KOPN-8 cells that were treated with immunotoxin for the indicated times by western blot and quantified. Shown are the background-subtracted and normalized ratios of cleaved/whole PARP over time. The graph summarizes the results from biological duplicates, each done in technical duplicates; symbols indicate mean values, errors as SEM.

To test whether biochemical events reflected their distinct activity in vitro, we analyzed the critical steps of the immunotoxin intoxication processes. First, we compared the rate of internalization using Alexa647-conjugated immunotoxins (Figure 4A). For the three variants, the mean fluorescence intensity (MFI) of Alexa647 increased almost linearly over 6 h, and there was no significant difference between the MFIs of any of the immunotoxins. We next determined the rate of protein synthesis over time of immunotoxin treated cells (Figure 4B). Correlating with the shorter needed exposure time to kill efficiently, the dsFv-PE38 reduced protein synthesis in an exponential manner, and much faster than PE24 and PE24(B), which reduced protein synthesis in a linear manner. The rate of arresting protein synthesis was not different for PE24 and PE24(B). Thus, the advantage of the domain II containing immunotoxin against KOPN-8 occurred after internalization, but before the arrest of protein synthesis. Additionally, the time to reach the late-apoptotic PARP-cleavage was determined (Figure 4C). Six hours after the treatment started, the ratio of cleaved over uncleaved PARP was 1.12 for dsFv-PE38, 0.48 for Fab-PE24, and 0.13 for Fab-PE24(B). The time that is needed to induce the late-apoptotic event of PARP-cleavage in KOPN-8 cells was the shortest after treatment with dsFv-PE38, longer after Fab-PE24, and the longest after Fab-PE24(B), thereby also confirming differences in the efficacy of inducing cell death biochemically.

### 2.5. Lack of Domain II Is Advantageous against Primary ALL Blasts

**Figure 5:**
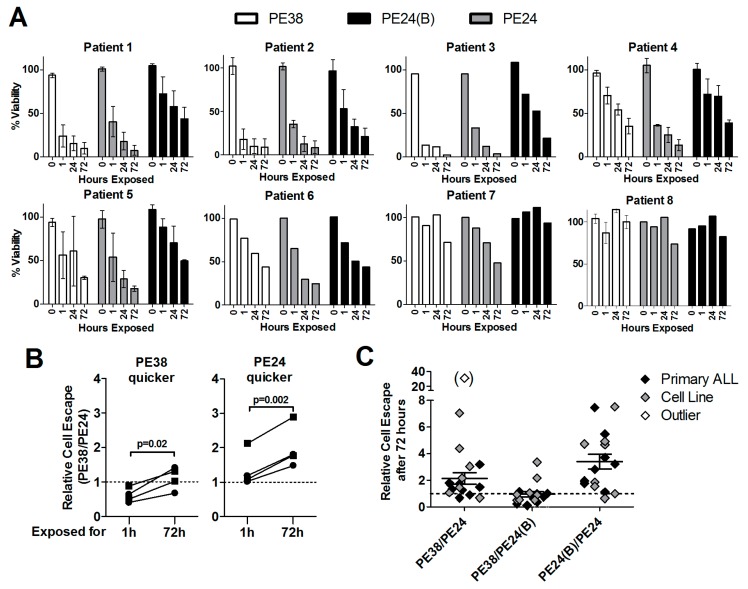
Patient-derived primary ALL cells are on average most sensitive to the PE24-variant. (**A**) Primary patient derived B-ALL cells were co-cultured on murine OP-9 stromal cells and treated with 2.8 nmol/L immunotoxin for the indicated times, washed, and replated on the same feeder layer. Three days after assay initiation, cell viability of the human ALL cells was determined by flow cytometry. Bars indicate the mean % of living cells at each time point, errors shown as SEM; and, (**B**) The relative % of cell killing was normalized as cell killing by PE24/cell killing by PE38 after 1 h and after 72 h of drug exposure. All of the cell lines were divided into two groups, one where PE24 was less active after only 1 h (PE38 quicker) and one where PE24 was more active after 1 h (PE24 quicker). Results of the same cell line after 1 h and after 72 h of exposure are connected by a line, p-values determined by paired *t*-tests; and, (**C**) Results from cell lines (grey rhombs) and patient cells (black rhombs) were pooled and the relative cell escape at 72 h of continuous exposure mathematically determined by dividing the fraction of living cells after the indicated treatment. The mean relative cell escape is indicated by a black line, error as SEM, clear rhomb in brackets was defined as outlier and was excluded from the mean relative escape.

Also, primary patient-derived ALL blasts had to be exposed to 2.8 nmol/L immunotoxin for a highly variable time for them to die (Figure 5A). More than 50% of ALL blasts of patients 1, 2, and 3 died after only 1 h of exposure to dsFv-PE38 or Fab-PE24, of patients 4–7 after at least 24 h, and 50% cell killing was not yet reached at 72 h of immunotoxin treatment for patient 8. As for KOPN-8 and Reh cells, blasts of patients 1, 2, 3, and 5 were killed more efficiently after shorter exposure to dsFv-PE38 than after exposure to Fab-PE24 or Fab-PE24(B). On the other hand blasts of patients 4, 6, 7, and 8 died more efficiently after the short exposure to Fab-PE24 than after short exposure to dsFv-PE38 or Fab-PE24(B). Comparing the relative cell escape showed that even though being slower initially, treatment with Fab-PE24 was more potent than dsFv-PE38 at later time points against patient-derived ALL blasts (Figure 4B). ALL blasts that showed less cell escape after 1 h of treatment with Fab-PE24 than after dsFv-PE38 consistently showed less escaping cells when being treated with Fab-PE24 for 72 h. On the other hand, three of four primary ALL-blasts that showed less relative cell escape after 1 h of treatment with dsFv-PE38 than with Fab-PE24 showed less escaping cells after 72 h of exposure to Fab-PE24 than to dsFv-PE38 at 72 h. We then compiled the data on relative cell escape of cell lines and patient samples after 72 h of continuous treatment (Figure 5C). After 72 h of continuous treatment, on average, 2.2 (±0.44) times more cells escaped treatment with dsFv-PE38 than with Fab-PE24, 3.40 (±0.55) times more cells escaped after PE24(B) than after PE24, and the relative cell escape was similar after dsFv-PE38 and Fab-PE24(B) (0.95 ± 0.20) demonstrating the highest overall-activity after 72 h of continuous treatment with the WT PE24 immunotoxin.

## 3. Discussion

Testing five immunotoxin variants, we determined which difference between dsFv-PE38 and the deimmunized variant Fab-PE24(B) was responsible for a 4000-fold difference in efficacy in a KOPN-8 xenograft model and found WT domain II of PE38 to be most important for the highest activity after bolus dose administration. In vitro, domain II shortened the time KOPN-8 cells had to be exposed to immunotoxin for them to die to a few minutes, while the lack of domain II combined with deimmunized domain III extended the time that is needed to kill cells to several hours. Because immunotoxin serum concentration falls quickly after intravenous bolus doses, the drug exposure time that is needed to induce cell death was the most critical to predict in vivo efficacy of bolus doses of immunotoxin, whereas the current method of determining cytotoxicity using three-day continuous treatment was not. Even though PE38 was most active in the KOPN-8 xenograft model when being administered as bolus doses, continuous exposure to any immunotoxin produced substantially higher rates of cell death in vitro. When continuously exposed, WT PE24 was on average more cytotoxic than both PE38 and PE24(B), suggesting that the PE24-variant may be most favorable when treating CD22-expressing malignancies continuously in vivo.

### 3.1. How Domain II Influences Immunotoxin Efficacy

All five immunotoxin variants were internalized at a similar rate, indicating that a similar number of immunotoxin molecules entered the cells, and thus, presumably the endosomal compartment. A crucial step of immunotoxin intoxication is endosomal escape of domain III which then ADP-ribosylates elongation factor 2 in the cytosol [16]. No method can directly measure immunotoxin trafficking currently. Because a defined number of PE-molecules needs to reach the cytosol to kill a cell [17], we believe that the transport of PE38 is more efficient than the transport of PE24 and of PE24(B). In line with more efficient trafficking, arrest of protein synthesis by PE38 with full domain II follows a much faster, exponential time course, while PE24 arrests protein synthesis slower and proportionally over time. The distinct kinetics may suggest an additional function of domain II beyond a furin cleavage site. Whether domain II plays a role in directed vesicular cargo-transport towards the Trans-Golgi network is possible, but it has not been previously described [18,19,20]. Furin-cleavage of domain II was shown to be the most efficacious at the acidic pH of late endosomes [21], suggesting that domain II may allow for a timely liberation of domain III, whereas the furin-cleavage site of PE24 may be accessible in a pH independent manner [12]. It was shown that domain II of dsFv-PE38 is cleaved more efficiently by furin than PE24(B) when being internalized into KOPN-8 cells [15], and that the alterations of domain II and the furin-cleavage site greatly influence cytotoxicity [22,23]. Intriguingly, CD22-targeted immunotoxins that cannot be cleaved by furin loose approximately three-fold cytotoxicity, whereas mesothelin-targeted immunotoxins that cannot be cleaved by furin are inactive, indicating that the effects of furin-cleavage on cytotoxicity is target receptor and target cell dependent [24]. This is in line with highly variable endosomal (toxin) transport [25,26], and with a substantial variability of PE-trafficking depending on the target receptor and the target cell type [27,28,29,30,31]. 

A cell type specific transport of domain II-containing immunotoxin is also in line with the more efficient killing of some ALL, but not lymphoma cells after the short exposure shown in this study and is further supported by a higher clinical response rate to bolus dose Moxetumomab of 34% in pediatric ALL [9], while the response rate in adult ALL is 13% [32] and adult lymphoma patients fail to respond to domain II containing immunotoxin [33]. We cannot currently predict which ALL patients may respond better to bolus doses of domain II-containing immunotoxin.

### 3.2. Efficacy In Vivo Is Influenced by Needed Exposure Time and Half-Life

Generally, larger Fab-containing immunotoxins needed a longer exposure time than dsFv-containing immunotoxins in vitro. However, in vivo, the larger Fab-variants showed two- to four-fold higher efficacies, which is likely due to the two-fold longer half-life of larger Fab-molecules [14]. That Fab-variants were less active in vitro, but more active in vivo, suggests that in vivo efficacy is predicted by (i) the needed exposure time to kill target cells and (ii) serum half-life. These data add to our novel understanding that exposure time is a crucial component of in vivo efficacy of immunotoxins [15,34]. To prolong serum half-life, novel immunotoxins containing an albumin-binding domain were constructed, which, in line with our results, show improved efficacy in vivo [35]. Whether smaller dsFv containing immunotoxins given continuously to compensate for the short half-life may be more efficacious due to improved tissue penetration [36,37] is a question of ongoing research.

In summary, the present study suggests that approximately 1/3 of ALL are killed better after short exposure to domain II containing PE than after short exposure to the PE lacking domain II. Cytotoxicity of CD22-targeted immunotoxins substantially increase the longer cells that are exposed, thus emphasizing that patients with B-cell malignancies may respond better to continuous rather than to bolus dose administration. After 72 h, PE24 is on average two-fold more cytotoxic than PE38 suggesting that PE24 may be more efficacious than PE38 when being administered continuously.

## 4. Materials and Methods

### 4.1. Reagents

The immunotoxins dsFv-PE38, also Moxetumomab pasudotox [38], dsFv-PE24, also LR [12], the dsFv-PE24(B), and the Fab-PE24(B) [14] were produced, as previously described. The immunotoxin Fab-PE24 was cloned by exchanging the dsFv of LR with the Fab of Fab-PE24(B). The resulting protein was produced following standard procedures [39].

Secondary western blot antibodies were purchased from Santa Cruz Biotechnology (Santa Cruz, CA, USA), primary antibodies (Actin, GAPDH, and PARP) from Cell Signaling (Danvers, MA, USA), flow cytometry antibodies, and Annexin V-PE/7-AAD from Becton Dickinson (Franklin Lakes, NJ, USA).

### 4.2. Cell Lines

The cell lines were described previously [15,34]. All of the cells were grown in RPMI, supplemented with 10% fetal bovine serum, 100 U penicillin, and 100 mg streptomycin (Invitrogen, Carlsbad, CA, USA).

### 4.3. Cell Assays

Cytotoxicity was determined by WST-8, as described [15]. 5000 Cells/well were incubated with various recombinant immunotoxin (rIT) concentrations for 72 h. WST-8 reagent was added, absorbance measured 2 h later, and the values were normalized between Cycloheximide (10 μg/mL final, Sigma-Aldrich, Saint Louis, MO, USA) and untreated control. Non-linear regression to obtain IC_50_ concentrations was done using GraphPad Prism v6.01.

For in vitro apoptosis assays by flow cytometry, 1 million cells/mL were incubated with 2.8 nmol/L immunotoxin for various times, cells were washed twice, and were transferred to a new plate in complete RPMI. Seventy-two hours after assay initiation, cells were stained with 7-AAD/Annexin-PE, and measured with a FACS Calibur (Becton Dickinson, Franklin Lakes, NJ, USA). Results were analyzed with FlowJo software (Tree Star, v10.2, St. Ashland, OR, USA, 2016).

For primary patient cell assays, 20,000 OP-9 stromal cells were plated per well in a 24-well plate in α-MEM (1% P/S, 20% FBS) on Day 0. On Day 1, 300,000 patient cells were added and 2.8 nM rIT 4 h later. At the indicated times, cells and wells were washed twice with PBS and the cells were re-plated in the same co-culture wells. Seventy-two hours after initiation, the cells were stained with anti-hu-CD19 and Annexin V-PE/7-AAD and analyzed by flow.

For internalization assays immunotoxins were conjugated with Alexa647, following the manufacturer’s instructions (Invitrogen). One million cells were incubated with Alexa647-conjugated immunotoxin at saturating concentrations (3 µg/mL) at 37 °C for the indicated times. Surface bound molecules were stripped for 10 min in 0.2 M Glycine pH 2.5, cells washed twice with phosphate buffered saline, and analyzed by flow cytometry (FACS Calibur, Becton Dickinson). 

Inhibition of protein synthesis was determined by [^3^H]-leucine incorporation, as described [12]. One million cells/mL were plated in complete RPMI and were treated for indicated times. Cells were pulsed with 2 µCi [^3^H]-leucine for 1 h, frozen on dry ice for 30 min, thawed at 37%, and CPM was read using a liquid scintillation counter.

### 4.4. Patient Samples

Primary ALL samples from eight patients with CD22-positive pre-B-ALL treated on a phase I trial of HA22 (NCT01891981) were collected with informed consent before the first HA22 dose under protocol 04-C-0102, and was approved by the NCI Institutional Review Board

### 4.5. Animal Studies

Animals were handled according to NIH guidelines; studies under protocol #LMB-063 were approved 08/04/2010 by the NCI Animal Care and Use Committee.

Five million KOPN-8 cells were injected on Day 1 via tail vein into 6- to 8- week-old NSG mice (NOD.Cg-*Prkdc^scid^ Il2rg^tm1Wjl^*/SzJ). Immunotoxin was given intravenously QOD at the maximal tolerated dose of 0.4 mg/kg (dsFv-PE38), 1 mg/kg (Fab-PE24), 2 mg/kg (dsFv-PE24), 2.5 mg/kg (Fab-PE24(B)), and 4 mg/kg (dsFv-PE24(B)). To assess early response, mice were euthanized three days after the last dose. For survival studies, mice were followed until disease progression (weight loss > 10%). BM was extracted by flushing femurs. Human ALL was stained with anti-human-CD19-FITC. Experiments on mouse-derived tissue were F_c_-receptor blocked with anti-murine CD16/32.

### 4.6. Statistics

Statistical analyses were performed using Graph Pad Prism v6.01 (La Jolla, CA, USA, 2012) as *t*-tests (two group comparison) or log-rank tests for animal survival as indicated.

## Supplementary Materials

The following are available online at http://www.mdpi.com/2072-6651/10/5/210/s1, Figure S1: Immunotoxin variants induce exposure time-dependent cell death, Figure S2: Immunotoxin variants indcue exposure time-dependent cell death, Table S1: IC_50_ pM for growth inhibition by WST8 assays, Table S2: 2-phase regression fitting of Figure 1.
