# Supplementary material for: Domain II of Pseudomonas Exotoxin Is Critical for Efficacy of Bolus Doses in a Xenograft Model of Acute Lymphoblastic Leukemia
Source: Toxins (Basel). 2018 May 21;10(5):210. doi: 10.3390/toxins10050210 (PMC5983266; doi:10.3390/toxins10050210)
Supplement: Supplementary file 1 [file toxins-10-00210-s001.pdf]

Supplementary Materials: Domain II of *Pseudomonas* exotoxin is critical for efficacy of bolus doses in a xenograft model of acute lymphoblastic leukemia

Fabian Müller, Tyler Cunningham, Richard Beers, Tapan K. Bera, Alan S. Wayne and Ira Pastan

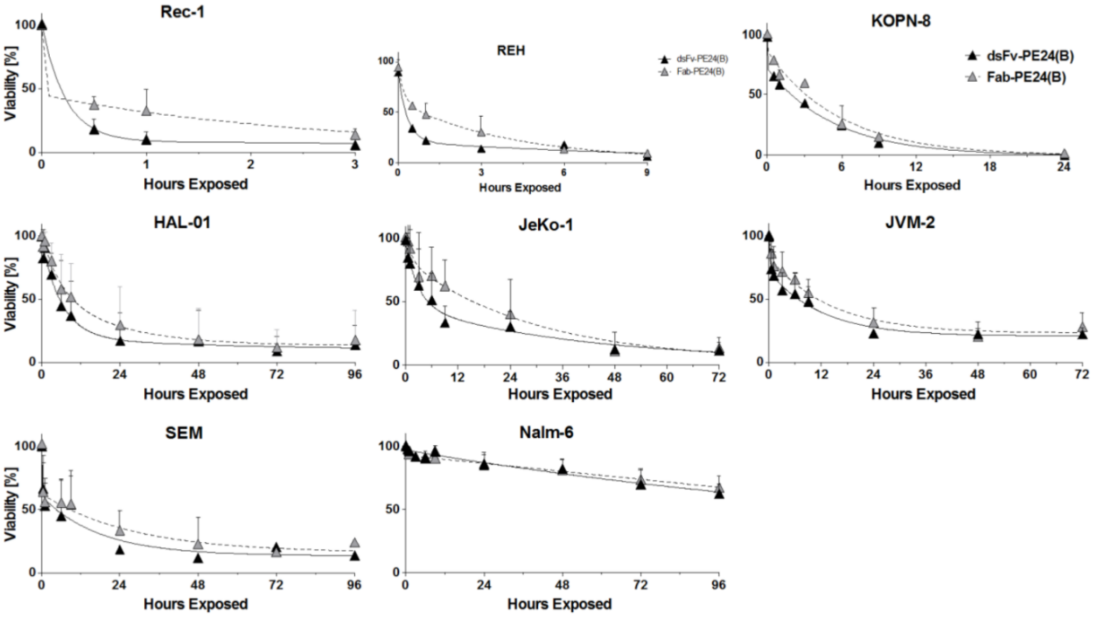

.

Figure S1. Immunotoxin variants induce exposure time-dependent cell death. Indicated B-cell lymphoma cell lines were treated with 2.8 nmol/L of the Fab and the dsFv variant of WT PE24 containing immunotoxin for the indicated times, washed, and replated. Three days after assay initiation, cell viability was determined by flow cytometry. The symbols indicate the mean % of living cells at each data point of at least three independent experiments, errors are shown as SEM, curve fitting was done using 2-phase decay regression analysis using GraphPad.

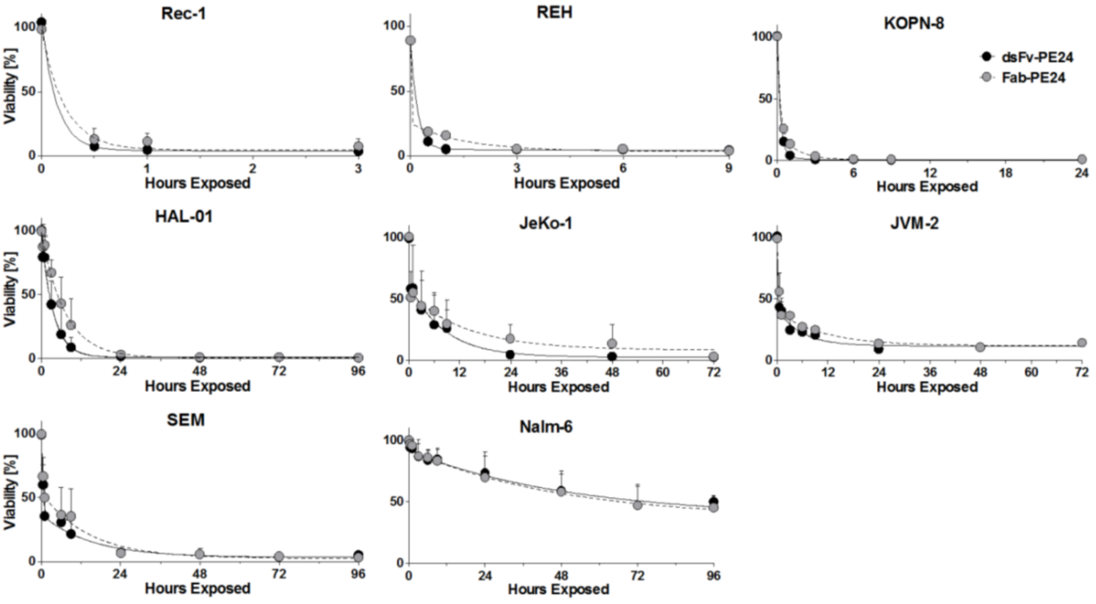

Figure S2. Immunotoxin variants indcue exposure time-dependent cell death. Indicated B-cell lymphoma cell lines were treated with 2.8 nmol/L of the Fab and the dsFv variant of the B-cell epitope depleted PE24(B) containing immunotoxin for the indicated times, washed, and replated. Three days after assay initiation, cell viability was determined by flow cytometry. The symbols indicate the mean % of living cells at each data point of at least three independent experiments, errors are shown as SEM, curve fitting was done using 2-phase decay regression analysis using GraphPad.

Supplementary Table S1. IC_{50} pM for growth inhibition by WST8 assays.

		Fv-PE38	Fv-PE24	Fab-PE24	dsFv-PE24(B)	Fab-PE24(B)
REH	(ALL)	0.3	0.9	1.6	2.2	2.9
KOPN-8	(ALL)	1.2	2.7	3.9	7.2	10.9
HAL-1	(ALL)	29.6	6.9	10.9	17.2	25.5
SEM	(ALL)	50.9	33.4	27.1	66.6	62.8
Nalm-6	(ALL)	32	19.2	21	45.6	54.9
Rec-1	(MCL)	5	3.8	10	6.2	9.3
JVM-2	(MCL)	19.2	9.8	16.2	28.5	34.8
Jeko-1	(MCL)	25.2	13.6	26.4	29.8	44.7
CA-46	(BL)	1.5	1.7	2.5	3.4	5.8
Median IC_{50}		19.2	**6.9**	**10.9**	17.2	25.5
Min		**0.3**	0.9	1.6	2.2	2.9
Max		50.9	33.4	27.1	66.6	62.8
Fold-difference		170	**37**	**17**	30	22
Variance (σ^2)		278	**100**	**88**	430	448

Values are average IC_{50}s in pM of at least 3 independent experiments. Cell lines are ranked by entitiy (ALL = acute lymphocytic leukemia, MCL = Mantle Cell Lymphoma, BL = Burkitt's Lymphoma) and subsequently by dsFv-PE38-cytotoxicity form lowest to highest IC_{50}.

Table S2. 2-phase regression fitting of Figure 1.

		**Fab-PE24(B)**	**Fab-PE24**	**dsFv-PE38**
HAL-1	Half Life (Slow)	15,21	4,933	25,97
	Half Life (Fast)	4,075	4,349	8,262
JeKo-1	Half Life (Slow)	20,31	9,566	14,26
	Half Life (Fast)	0,965	0,007186	0,001893
JVM-2	Half Life (Slow)	9,621	7,59	9,16
	Half Life (Fast)	0,2264	0,2548	0,2457
KOPN-8	Half Life (Slow)	4,015	0,9565	0,0576
	Half Life (Fast)	0,0002076	0,157	4,48E-05
Nalm-6	Half Life (Slow)	126,4	33,85	95,64
	Half Life (Fast)	51,67	0,8728	45,52
Rec-1	Half Life (Slow)	1,737	3,71	5,349
	Half Life (Fast)	0,001111	0,001075	0,1638
Reh	Half Life (Slow)	2,802	1,043	0,2193
	Half Life (Fast)	0,1464	0,005527	0,001013
SEM	Half Life (Slow)	17,44	8,17	6,985
	Half Life (Fast)	0,1401	0,2749	0,1632

Shown are all cell lines from Figure 2 in alphabetical order. The time, cells had to be exposed was determined experimentally and analyzed with a two-phase regression model. Thus, two half-lives are determined as fast and slow for the respective immunotoxin.
